# Antioxidant and Antimicrobial Activities of Thai Edible Plant Extracts Prepared Using Different Extraction Techniques

**DOI:** 10.3390/molecules27196489

**Published:** 2022-10-01

**Authors:** Pimmada Junsathian, Soichiro Nakamura, Shigeru Katayama, Saroat Rawdkuen

**Affiliations:** 1 Food Science and Technology Program, School of Agro-Industry, Mae Fah Luang University, Chiang Rai 57100, Thailand; 2 Institute for Biomedical Sciences, Interdisciplinary Cluster for Cutting Edge Research, Shinshu University, 8304 Minamiminowa, Nagano 399-4598, Kamiina, Japan; 3 Unit of Innovative Food Packaging and Biomaterials, School of Agro-Industry, Mae Fah Luang University, Chiang Rai 57100, Thailand

**Keywords:** antioxidative activity, antimicrobial activity, Thai edible plant, microwave-assisted extraction, ultrasonic-assisted extraction

## Abstract

This study investigated the antioxidant and antimicrobial activities of six Thai edible plant leaf extracts, including Cashew (CN), Chamuang (CM), Monpu (MP), Thurianthet (TT), Kradon (KD) and Pakliang (PL), extracted using ethanol extraction (EE), microwave-assisted extraction (MAE), and ultrasonic-assisted extraction (UAE). The leaf extracts were characterized for percentage yield, total phenolic content (TPC), total flavonoid content (TFC), 2,2-diphenyl-1-picrylhydrazyl (DPPH) and-ferric reducing antioxidant power (FRAP) activity, and antimicrobial activity against spoilage. MAE produced the highest percentage yields, among which MAE-extracted MP exhibited the highest yield. Furthermore, the highest TPC and TFC were obtained for MAE, with MAE-extracted KD and CN showing the highest TPC and TFC, respectively, among the samples. The highest DPPH and FRAP values were seen in MAE-processed CN, KD, and MP extracts. The inhibition zone of pathogenic bacteria, minimum inhibitory concentration, and minimum bacterial concentration were determined in all samples except TT. These findings indicate that, compared to EE and UAE, MAE improved the antioxidant and antimicrobial efficacy of the leaf extracts. The aforementioned extracts could be employed as natural food additives to prevent chemical and microbial spoilage of foods.

## 1. Introduction

Plants are widely used for dietary and medicinal purposes in several parts of the world. Bioactive compounds from various plants benefit human health by providing protection and nutrition, as well as protection against microorganisms [1,2]. Recently, many researchers have shown interest in identifying the antioxidative properties and antimicrobial efficacy of plant materials [3,4]. Bioactive compounds found in fruits and vegetables are secondary metabolites classified as a subclass of phytochemicals with biological activities, and plant polyphenols are potential antioxidants for human health [5]. Antioxidants can reduce oxidative damage by neutralizing free radicals and eliminating them from the body [6]. Several epidemiological studies have revealed that plant polyphenols reduce the risk of chronic human disease [7,8]. 

In addition to antioxidative activities, plant polyphenols serve as preservatives in food processing. Several studies have shown that antioxidant substances can act in various ways, including as free radical scavengers or chelators, preventing lipid oxidation and thereby preventing nutrient losses and inhibiting the potential formation of toxic compounds [9,10]. Food spoilage and food poisoning caused by the growth of pathogenic bacteria are major issues in the food industry. There is a growing interest in using natural active preservatives, particularly plant extracts, to prevent food spoilage and preserve foods without posing health risks [11]. 

The antioxidant and antimicrobial activities of various plant polyphenols have been studied extensively and are used in various food, cosmetic, and pharmaceutical applications [12,13]. The bioactivity of plant-derived extracts containing high amounts of polyphenols depends on their efficiency of extraction [14]. Novel extraction techniques have several benefits over conventional methods, such as excellent recovery and improved stability and quality of the extract [15]. According to Dahmoune, et al. [16], microwave-assisted extraction (MAE) and ultrasonic-assisted extraction (UAE) can increase plant polyphenol yields under controlled time and temperature conditions. UAE is a novel green chemistry method that allows for the extraction of bioactive compounds with low solvent and energy costs, while preserving the integrity of the extracted molecules [17]. MAE is a process that extracts the target compounds from various matrices using microwave energy and extraction solvents. Aqueous ethanol is more effective than absolute ethanol for the extraction of bioactive compounds because it improves the extractability of the compounds by increasing the polarity of the extraction solvent.

Thailand is home to several edible plant species that can grow and survive in tropical zones. Thai people use these plants in various traditional diets, dishes, and medicinal beverages. Six edible Thai plants, including Cashew (*Anacardium occidentale* L.) (CN), Chamuang (*Garcinia cowa* Roxb.) (CM), Monpu (*Glochidion wallichianum* Muell Arg.) (MP), Thurianthet (*Annona murica*) (TT), Kradon (*Careya sphaerica* Roxb.) (KD), and Pakliang (*Gnetum gnemon* var. Temerum) (PL), were studied to compare the efficacy of MAE, UAE, and the conventional ethanol extraction (EE), particularly in terms of enhancement of extraction yield and bioactive properties. This is the first comparative study of edible Thai plant extracts extracted using different extraction methods. The purpose of this study was to compare the efficacy of two novel extraction techniques (UAE and MAE) with that of the conventional EE technique on the total phenolic and flavonoid yield, as well as on the antioxidant and antimicrobial activities of various Thai leaf extracts.

## 2. Results and Discussion

### 2.1. Extraction Efficiency of MAE, UAE, and EE

**Figure 1:**
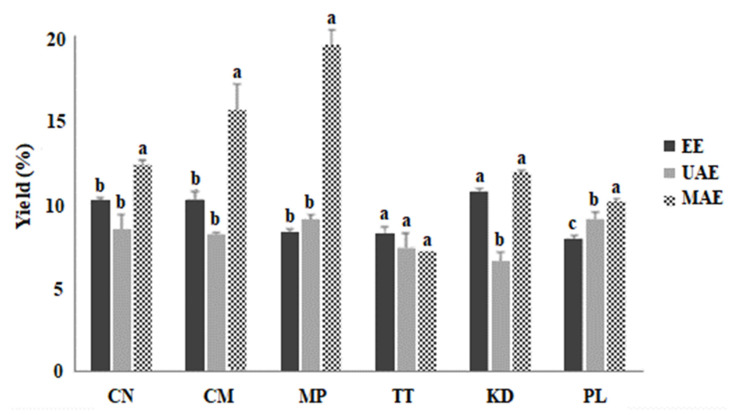
Percentage yield of edible Thai plant extracts by different extraction techniques. Values are presented as the mean ± standard deviation (SD) of triplicate analyses (*n* = 3). Different letters (a–c) denote significant differences (*p* < 0.05) between mean values of the extraction method for each plant material. CN; Cashew, CM; Chamuang, MP; Monpu, TT; Thurianthet, KD; Kradon, PL; Pakliang, EE; ethanol extraction, UAE; ultrasonic-assisted extraction, MAE; microwave assisted extraction.

Figure 1 depicts the extraction yield of six Thai plant extracts prepared using EE, UAE, and MAE, with 60% ethanol. The results clearly demonstrate that MAE produced the highest yield compared to the other extraction methods (*p* < 0.05). This may be because the heat generated within the materials affects the heating kinetics and increases the pressure within the cells. These effects could degrade the plant cell wall structure faster and more easily than the other methods, resulting in a higher diffusion rate of the solvent, and thereby increasing the extraction yield [18]. This indicates that MAE is more efficient than the other two methods in increasing the extraction yield. The highest percentage yield was obtained for MP extracted via the MAE method (*p* < 0.05). UAE showed lower extraction yields (*p* < 0.05) than EE and MAE. Polyphenol extraction from carob (*Ceratonia siliqua*) kibbles using a microwave-assisted technique resulted in a high yield [18].

### 2.2. Total Phenolic and Flavonoid Contents of Plant Extracts Prepared by MAE, UAE, and EE Processes

**Figure 2:**
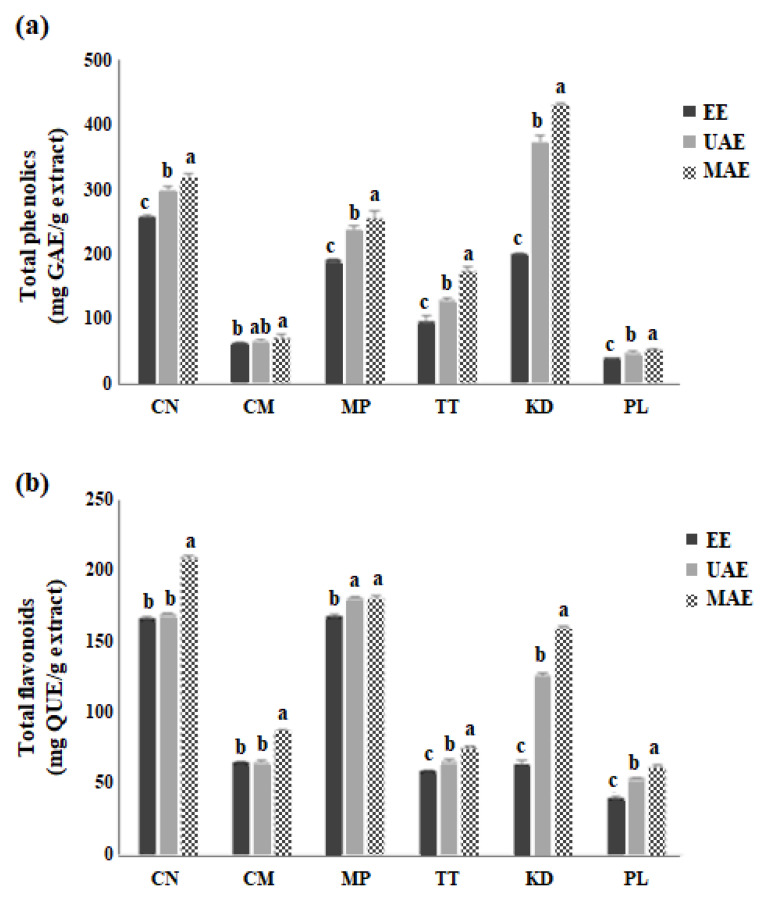
Total phenolic (**a**) and total flavonoid contents (**b**) of six Thai edible plant extracts extracted using EE, UAE, and MAE. The data are presented as the mean ± SD of triplicate analyses (*n* = 3). Different letters (a–c) denote significant differences (*p* < 0.05) between extraction methods for each plant. GAE, gallic acid equivalent; QUE, quercetin equivalent.

The total phenolic content (TPC) and total flavonoid content (TFC) of the six plant extracts are shown in Figure 2a. MAE produced the highest TPC for each extracted plant material, followed by UAE and EE. MAE and UAE methods were more efficient than the conventional EE (*p* < 0.05). Amongst the extracts, MAE-processed KD showed the highest TPC (*p* < 0.05). Meanwhile, the TPCs obtained by EE were the lowest among the three methods (*p* < 0.05). The CM and PL extracts had the lowest TPC, regardless of the extraction process, compared to the other plant materials. This may be due to the low polyphenol content in their plant cell matrices. MAE has been reported to increase the TPC of plant materials during the extraction process compared with ultrasonic-assisted pressurized liquid extraction and EE [19]. 

The MAE-extracted samples showed the highest TFC compared with the UAE- and EE-extracted samples (*p* < 0.05) (Figure 2b). Among the MAE-extracted samples, the CN, MP, and KD showed the highest amount of flavonoids (207.7 ± 1.3, 179.1 ± 1.7, and 158.6 ± 1.2 mg QUE/g dry extract, respectively). The highest TFC was observed in MAE-extracted CN, extracted using 60% ethanol. The high TFC obtained via MAE can be associated with the heat generated due to the interaction of microwaves with polar molecules within the plant cells, which creates high pressure to disrupt the cell wall. After cell wall disruption, the diffusion of solvent into plant cells leads to the extraction of higher amounts of phenolic substances [20]. The MAE technique has been documented to obtain higher levels of flavonoids and phenolics from *Herba Epimedii* and ginger [21,22]. UAE works via the cavitation effect, by creating bubbles in the extraction solvent which burst and produce high flashes of energy and thereby disrupt the cell wall of the plant material [23]. Ethanol (60%) also affects the yield of phenolic substances owing to its polarity, as well as water content, which enhances the extraction efficiency by extracting hydrophilic bioactive compounds [24]. In this study, the powder samples of leaf were used to obtain extracts using EE, UAE, and MAE systems; however, the particle size was not measured in these experiments. Further investigations will be needed to assess the extraction efficiency of different particle size samples.

### 2.3. Impact of MAE, UAE, and EE on the Antioxidant Activity of Plant Extracts

**Figure 3:**
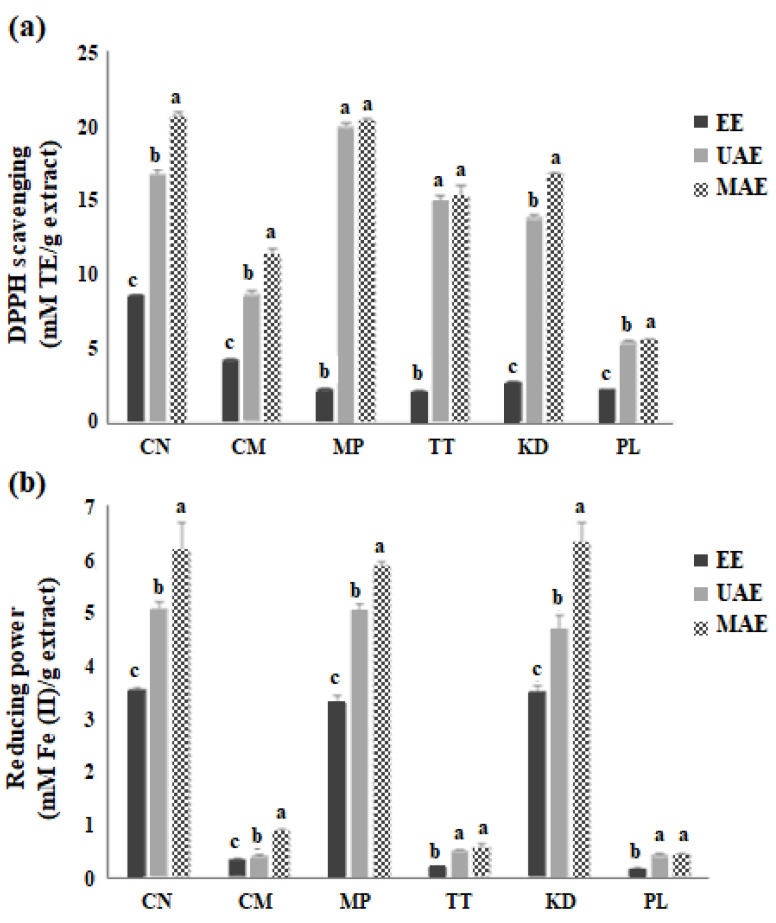
The antioxidant activity of six Thai edible plant extracts extracted by the conventional EE, UAE, and MAE; 2,2-Diphenyl-1-picrylhydrazyl (DPPH) scavenging (**a**) and ferric-reducing antioxidant power (FRAP) (**b**). The data are presented as the mean ± SD of triplicate analyses (*n* = 3). Different letters (a–c) on the bars denote significant difference (*p* < 0.05) of means between extraction methods for each plant. TE; Trolox equivalent.

The 2,2-Diphenyl-1-picrylhydrazyl (DPPH) and ferric-reducing antioxidant power (FRAP) activities of the Thai plant extracts were investigated for antioxidant activity, as shown in Figure 3. All samples extracted via the MAE process exhibited higher DPPH radical-scavenging activity (*p* < 0.05) compared to the samples extracted using UAE and EE. Irrespective of the extraction method, CN, KD, and MP presented the highest values of DPPH activity, with CN extracted via MAE having the highest value (20.6 ± 0.2 mM Trolox equivalent (TE)/g extract), as shown in Figure 3a. MAE-extracted MP showed the second-highest DPPH value (20.23 ± 0.1 mM TE/g extract). The FRAP results were also in line with the DPPH activity, with MAE-extracted KD, CN, and MP exhibiting stronger antioxidant power than the other samples with values of 6.5 ± 0.3, 6.3 ± 0.5 and 6.1 ± 0.1 mM Fe (II)/g extract, respectively (Figure 3b). Based on the above values, the highest FRAP activity was observed for MAE in CN and KD, followed by MP, compared to that obtained by the conventional EE process (*p* < 0.05). Although lower than the values obtained for MAE, the UAE processed samples also showed higher antioxidant activity when compared to EE (*p* < 0.05), irrespective of the individual activities of the sample extracts. PL showed the lowest DPPH and FRAP activities regardless of the extraction method used. 

Zhang, et al. [25] demonstrated that flavonoids extracted from *Epimedium sagittatum* using MAE showed the highest antioxidant activity, yield percentage, and chemical composition. Moreover, UAE was found to optimize the antioxidant activity of phenolic compounds extracted from peaches and pumpkins [26]. It is reasonable to assume that a positive relationship exists between antioxidant assays (DPPH and FRAP) and phenolic compounds, because high amounts of phenolic compounds exhibited strong antioxidant activity by inhibiting free radicals and electron donation from the phenol groups [27]. As expected, the enhanced extraction of phenolic and flavonoid contents by MAE resulted in an increase in antioxidant activity. Therefore, DPPH free radical scavenging and FRAP involving electron transfer reactions are important parameters for predicting the bioactivity of plant extracts [28]. 

### 2.4. Disc Diffusion Test, MIC, and Minimum MBC of Thai Edible Plant Extracts Prepared by MAE, UAE, and EE

**Figure 4:**
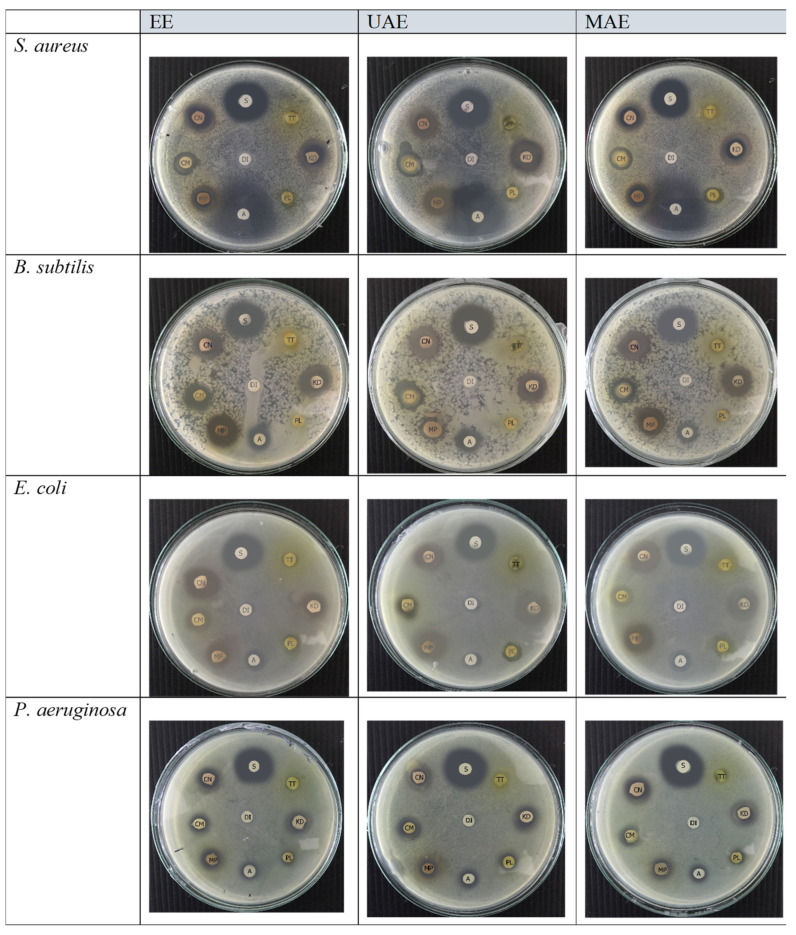
Zone of inhibition of plant extracts prepared via MAE, UAE, and EE against Gram-positive (*Staphylococcus* *aureus* and *Bacillus subtilis*) and Gram-negative (*Escherichia coli* and *Pseudomonas aeruginosa*) bacteria assessed by the disc-diffusion method. Streptomycin (S), ampicillin (A), and deionized water (DI) (30 µg/mL) were used as positive controls. Paper disc diameter, 6 mm.

The inhibition zones of the extracts prepared by MAE, UAE, and EE against the four selected pathogenic bacterial strains are shown in Figure 4 and summarized in Table 1. Most of the extracts showed antimicrobial activity with inhibition zones ranging from 14 to 17 mm for Gram-positive and 9 to 16 mm for Gram-negative bacterial strains, except for the TT extract, which revealed no microbial inhibition. Gram-positive bacteria were more sensitive to the extracts than Gram-negative bacteria, possibly because of the thickness of the cell membrane, which permits the passage of bioactive compounds more easily [29]. Although not observed in our study, a previous study reported that TT has antimicrobial properties. Haro, et al. [30] reported that the methanol extract of soursop (*Annona muricata* L.) leaves showed effective inhibition against *S. aureus* and *E. coli* at concentrations of 150 and 250 mg/mL, respectively.

The Thai plant extracts were analyzed for MIC and MBC, and the results are presented in Table 2. All plant extracts were effective against Gram-positive bacterial strains, including *S. aureus* and *B. subtilis*, with MIC ranging from 0.78 to 50 mg/mL and MBC ranging from 12.5 to 50 mg/mL. For Gram-negative strains, the MIC values ranged from 1.56 to 50 mg/mL. The lowest MIC values against Gram-positive bacteria were obtained for the MP and KD extracts prepared using MAE and UAE. The KD sample was also effective against Gram-negative strains with the lowest MIC values, along with CN (1.56 mg/mL). Additionally, the MBC values of the selected plant extracts for Gram-positive and Gram-negative strains ranged from 12.5 to 50 mg/mL and 25 to 100 mg/mL, respectively, indicating marked inhibition of the tested bacteria with the lowest bacterial counts. The lowest MBC against *S. aureus* was obtained for the CN sample extracted using MAE (12.5 mg/mL). MAE-extracted CN, MP, and KD showed the lowest MBC values for *B. subtilis* (25 mg/mL). This indicated that the Gram-positive strains were more sensitive to the CN, MP, and KD extracts. 

MIC and MBC values were obtained for all plant extracts, except TT. According to Solomon-Wisdom, et al. [31], methanolic and aqueous extracts of TT leaves inhibited *S. aureus, E. coli,* and *P. aeruginosa* at a concentration of 200 mg/mL. The extract obtained from KD was found to be a strong antimicrobial agent against both Gram-positive and Gram-negative bacterial strains, with the lowest MIC values compared to other extracts. A similar finding was observed by Daduang, et al. [32] who reported that the extract of the edible part of *Careya sphaerica Roxb*. (Kradon), which had high phenolic and antioxidant contents, inhibited *S. aureus, S. typhimurium,* and *E. coli*. Additionally, Chanudom, et al. [33] reported that the ethanol crude extracts of traditional Thai plants, including CN and MP, possess high phenolic and antioxidant content and are effective against *S. aureus, E. coli,* and *P. aeruginosa*.

There is considerable interest regarding the antioxidant activity of natural plant polyphenols against free radicals. In addition, in some cases, these substances also serve as active compounds against microorganisms such as bacteria, viruses, and fungi. Flavonoids possess antimicrobial activity because of their ability to interact with and disrupt extracellular soluble proteins and bacterial cell wall [1]. Some plant phenolic compounds, such as chlorogenic acid, curcumin, ellagic acid, (-) epicatechin, rutin, and tannic acid, were found to control the growth of foodborne pathogenic strains, including *Bacillus, Listeria,* and *Clostridium* species [34]. Jarriyawattanachaikul, et al. [35] reported the antimicrobial activity of Thai herbal plants against various foodborne pathogens (*E. coli*, *S. aureus*, and *Campylobacter* *jejuni*). Thai herbal extracts have been reported to be effective against both Gram-negative and Gram-positive bacteria. Moreno, et al. [36] reported on the mechanisms associated with the interaction between active phytochemical groups and cellular enzymes. The rate of penetration of bioactive compounds into microbial cells depends on membrane permeability, and disruption of cell membranes may lead to cell death due to the loss of cellular integrity. The water extract of rosella calyx (*Hibiscus sabdariffa*) extracted via the MAE method showed a higher zone of inhibition against *S. aureus* (Gram-positive bacteria) and *E. coli* (Gram-negative bacteria) [37]. MAE provides high extraction efficiency with minimum solvent requirements. Under microwave irradiation, the plant matrix is easily impregnated with solvents with higher heating efficiency. The higher temperature attained by microwave irradiation can hydrolyze ether linkages of cellulose, which are the main constituent of the plant cell wall, and can convert into soluble fractions within short period of time [38].

Costa et al. [39] demonstrated that the leaf extract of CN contained phytochemicals such as gallic acid and derivatives, quercetin derivatives, and luteolin using UPLC-DAD/QTOF-MS analysis. Phukhatmuen et al. [40] reported that 11 compounds including a novel decahydro-1H-xanthene derivative were identified from CM leaf extract, and most compounds were xanthones and benzophenones. In contrast, there are few studies focused on the separation and identification from the leaf extract of MP, TT, KD, and PL. Future studies will be needed to identify active phytoconstituents responsible for antioxidant and antimicrobial activities of these leaf extracts. 

## 3. Materials and Methods

### 3.1. Materials

Edible Thai plants, including CN, CM, MP, TT, KD, and PL, were collected from Chiang Rai and other Thai provinces. The leaves of these plants were washed with distilled water, ground into fine powder in liquid nitrogen using a stainless blender, and stored in a freezer at −20 °C until sample extraction. Folin–Ciocalteu reagent, Trolox, gallic acid, quercetin, dimethyl sulfoxide (DMSO), and streptomycin sulfate were purchased from Sigma-Aldrich (St. Louis, MO, USA). Nutrient broth and Mueller-Hinton broth (MHB) were purchased from HIMEDIA (Mumbai, India). Ampicillin and sodium chloride were purchased from Bio Basic (Markham, ON, Canada). Bacterial cultures were obtained from the Thailand Institute of Scientific and Technological Research. All the chemicals were of analytical grade.

### 3.2. Preparation of Thai Edible Plant Extracts Using the Conventional EE, UAE, and MAE 

Plant extracts were prepared from six Thai edible leaves using the conventional EE method, as described by Nagappan [41]. In brief, powdered leaf samples were mixed with 60% (*v*/*v*) ethanol at a ratio of 1:30 (g/mL of powder to solvent ratio) and placed in a shaker bath (Memmert, Germany) at 25 °C for 60 min. The UAE method was adapted from a previous study by Kazibwe, et al. [42]. In brief, the powdered leaf samples were mixed with 60% (*v*/*v*) ethanol at a ratio of 1:30 (g/mL of powder to solvent ratio) and sonicated using an ultrasonic bath (DT 255 H, Bandelin Co., Germany) at 25 °C for 60 min at a frequency of 35 kHz. The MAE method was adapted from Dahmoune, Nayak, Moussi, Remini and Madani [16] and Piovesan, et al. [43]. For this, a microwave oven (Samsung, Korea) was used as the heat source to facilitate extraction. Powdered leaf samples from the six Thai edible plants were mixed with 60% (*v*/*v*) ethanol at 1:30 (g/mL of powder to solvent ratio) and subjected to irradiation at 500 W for 60 s. After extraction using the three methods, the extracted samples were collected and filtered using filter paper (Whatman No.4) placed on a Buchner funnel equipped with a vacuum pump and concentrated using a rotary evaporator (IKA/RV 10 basic, Germany) at 45 °C for 10 min. The concentrated extracts were dried in a freeze-dryer (FD 8–55, Australia) at −50 °C for 72 h. The plant extracts were then stored at −20 °C in an airtight container prior to the determination of bioactive chemicals and microbial characteristics. 

### 3.3. Determination of Extraction Yield and Chemical Properties of Thai Edible Plant Extracts

Extraction yield

Percentage yield of the freeze-dried plant extracts was determined using the following equation:

Extraction yield (%) = gram of freeze-dried plant extract × 100/ gram of leaf powder

Determination of TPC and TFC

The TPC of Thai edible plant extracts was determined using the Folin–Ciocalteu assay (ISO 14502-1, 2005), and gallic acid was used as a standard. Samples of the extracts (500 μL each) were mixed with 2.5 mL of 10% (*w*/*v*) Folin–Ciocalteu reagent and 2 mL of 7.5% (*w*/*v*) sodium carbonate. The mixture was stirred and incubated in darkness for 1 h at room temperature (25 °C). The absorbance of the samples was measured at 765 nm using a microplate spectrophotometer (Thermo Fisher Scientific, Multiskan GO, USA) [44]. The TPC was expressed as milligrams of gallic acid equivalent (mg GAE)/g of dry extracts [45]. The TFC of the plant extracts was determined using the aluminum trichloride method, as reported by Rebaya, et al. [46], with some modifications. In brief, 36 µL of 5% NaNO_2_) and 45 µL of diluted sample were transferred to a 96-well microplate and left for 6 min. Next, 36 µL of 10% aluminum chloride hexahydrate was added to each well and the mixtures were incubated for 5 min. Thereafter, 180 µL of 10% sodium hydroxide was added and the final volume was adjusted to 300 µL using distilled water. The mixtures were incubated for another 15 min and the absorbance was measured at 510 nm using a microplate reader (Multiskan GO, USA). The TFC was expressed as milligrams of quercetin equivalent (mg QUE)/g of the extract.

Determination of DPPH and FRAP Activities

The free-radical-scavenging activities of Thai edible plant extracts were analyzed using the DPPH method [47]. First, the DPPH solution (60 mM) was prepared by dissolving 0.00236 g of 2,2-diphenyl-1-picrylhydrazyl in 95% ethanol (*v*/*v*). The solution was then mixed with the plant extracts (50 μL). Trolox (10,000 μM) was used as the standard solution and methanol was used as a blank. The mixtures were incubated at room temperature for 30 min. Absorbance was measured at 517 nm using a microplate spectrophotometer (Thermo Fisher Scientific, Multiskan GO, USA) [44]. DPPH activities of the plant extract samples were expressed as µmol TE/g of dry extract [48]. FRAP was evaluated using the method described by Sadeghi, et al. [49], with some modifications. Diluted plant extracts (35 µL) were pipetted into a 96-well microplate, and 265 µL of freshly prepared FRAP reagent (pre-incubated at 37 °C for a few minutes) was added to each well. Ethanol was used as a blank sample. The mixtures containing the plant extracts and FRAP reagent were incubated for 30 min, and the absorbance was measured at 595 nm. The FRAP activities of all samples were calculated based on the standard curve of ferrous sulfate (FeSO_4_·7H_2_O) in the range of 100 to 1000 µM. Activity is expressed as mM ferrous sulfate equivalent (mM Fe (II)) per gram of the extract. 

### 3.4. Determination of Antimicrobial Properties of Edible Thai Plant Extracts

The antimicrobial activity of the Thai plant extracts was evaluated using four bacterial strains. The bacterial strains were obtained from the Thailand Institute of Scientific and Technological Research (TISTR). Two strains of Gram-positive bacteria, *Staphylococcus aureus* TISTR 746 and *Bacillus subtilis* TISTR 008, and two strains of Gram-negative bacteria, *Escherichia coli* TISTR 527 and *Pseudomonas aeruginosa* TISTR 1278, were used to prepare the bacterial suspensions. 

Bacterial Suspension Preparation and Disk Diffusion Method

All the bacterial strains were activated in MHB for 18–24 h and sub-cultured overnight at 37 °C in MHB medium. The activated bacterial strains were diluted in 5 mL 0.85% sterile saline solution. Microbial suspensions were standardized according to a 0.5 McFarland scale (assumed cell count density 1.5 × 10^8^ CFU/mL) at 625 nm. The zone of inhibition was determined by the disc diffusion method to test the antimicrobial efficacy of the plant extracts, as described by Bauer, et al. [50] with some modifications. Bacterial suspensions of all strains (0.5 mL) were mixed with 25 mL of MHB and bacteriological agar. The agar medium containing the MHB-inoculated bacterial suspensions was poured into sterile Petri dishes and solidified for a few minutes. The disc diffusion method was performed by dropping 20 µL each of the sample extracts prepared in 20% DMSO (100 mg/mL) on a disc of 6 mm diameter and left in a laminar air flow for a few minutes before the incubation process. Distilled water and antibiotics (streptomycin and ampicillin) were used as the negative and positive controls, respectively. The plates containing the plant extracts, control (added with distilled water), and antibiotics were incubated for 12 to 24 h at 37 °C. The inhibition zone was then measured and expressed in millimeters.

Determination of MIC and MBC

The antimicrobial activity of edible Thai plant extracts was studied using a microdilution method adopted from Mosaddik, et al. [51]. Two Gram-positive (*S* *aureus* and *B. subtilis*) and two Gram-negative bacteria (*E. coli* and *P. aeruginosa*) were tested. The inoculum was prepared from a subculture using Muller-Hinton agar. Plant extracts were dissolved in 20% DMSO (100 mg/mL) and filtered using a 0.45 µm filter. Fifty microliters of each sample was diluted with 50 µL of MHB (50 mg/mL). The samples were then diluted two-fold in a 96-well microplate to attain a concentration range of 1.56 to 50 mg/mL. Bacterial strains without extracts were used as negative controls. Each well was inoculated with 5 µL of bacterial suspension (1 × 10^7^ CFU/mL). Inoculated plates were incubated at 36 °C for 18–24 h. Bacterial growth was then measured using a microplate reader at 625 nm. MIC values were determined as the lowest concentration of each extract which inhibited microbial growth. The results were expressed as milligrams per milliliter. A positive control (only cells plus medium) and negative control (only sample plus medium) were prepared for each tested bacterial strain. 

### 3.5. Statistical Analysis

All experiments were conducted in triplicate (*n* = 3). Data are expressed as the mean ± standard deviation. The IBM SPSS Statistics software ver. 23 was used for analysis of variance (ANOVA), as well as Duncan’s multiple range tests. *p*-values of less than 0.05 were considered statistically significant.

## 4. Conclusions

MAE, UAE, and the conventional EE process were employed to prepare phytochemical extracts from six edible Thai plant leaves. CN, CM, MP, TT, KD, and PL samples were processed using 60% ethanol, followed by MAE or UAE. The extraction yield was highest in the MAE-extracted MP leaf extract compared to the UAE and EE methods. TPC and TFC, including DPPH and FRAP values, were higher in the MAE-extracted KD and CN samples. MIC and MBC, including zones of inhibition against Gram-positive and Gram-negative bacterial strains, were highest in all MAE-processed plant samples except TT, which possibly required higher levels of the extract. Therefore, MAE is a promising method for extracting bioactive compounds using aqueous ethanol as an extraction solvent to enhance yield, polyphenol content, and antioxidant properties. MAE of plant extracts will be further investigated in the future for the isolation and characterization of active compounds and their fortification as natural food additives in perishable foods. 

## Figures and Tables

**Table 1:** Inhibition zones of Thai edible plant extracts against pathogenic bacterial strains obtained by the disc-diffusion test.

Bacterial Strains	Samples	Zone of Inhibition (mm)				
		EE	UAE	MAE	Ampicillin	Streptomycin
*S. aureus*					29	25
	CN	16	14	17		
	CM	14	14	14		
	MP	16	14	14		
	TT	NI	NI	NI		
	KD	17	17	18		
	PL	10	10	11		
*B. subtilis*					12	23
	CN	15	16	16		
	CM	14	15	15		
	MP	15	16	16		
	TT	NI	NI	NI		
	KD	17	17	17		
	PL	NI	NI	NI		
*E. coli*					13	22
	CN	12	14	15		
	CM	11	11	11		
	MP	10	15	15		
	TT	NI	NI	NI		
	KD	11	15	16		
	PL	9	10	11		
*P. aeruginosa*					12	26
	CN	14	15	16		
	CM	12	13	12		
	MP	13	13	14		
	TT	NI	NI	NI		
	KD	13	14	16		
	PL	9	10	10		

The concentrations of all sample extracts were 100 mg/mL and a volume of 20 µL was pipetted onto each paper disc (diameter 6 mm). NI, no inhibition.

**Table 2:** MIC and MBC of edible Thai plant extracts obtained via MAE, UAE, and EE processes.

Bacterial Strains	Plant	MIC (mg/mL)			MBC (mg/mL)		
		EE	UAE	MAE	EE	UAE	MAE
*Staphylococcus aureus*	CN	1.56	<1.56	0.78	25	25	12.5
	CM	3.12	<1.56	0.78	25	25	25
	MP	<1.56	<1.56	0.78	25	25	25
	TT	N/D	N/D	N/D	N/D	N/D	N/D
	KD	<1.56	<1.56	<1.56	25	25	25
	PL	50	50	12.5	50	50	50
*Bacillus subtilis*	CN	1.56	<1.56	<1.56	50	50	25
	CM	1.56	<1.56	<1.56	50	50	50
	MP	<1.56	<1.56	<1.56	50	50	25
	TT	N/D	N/D	N/D	N/D	N/D	N/D
	KD	<1.56	<1.56	<1.56	50	50	25
	PL	25	12.5	6.25	50	50	50
*Escherichia coli*	CN	1.56	1.56	1.56	50	50	25
	CM	12.5	12.5	12.5	100	100	100
	MP	6.25	3.12	3.12	50	50	50
	TT	N/D	N/D	N/D	N/D	N/D	N/D
	KD	3.12	3.12	1.56	50	50	50
	PL	50	50	50	100	100	100
*Pseudomonas aeruginosa*	CN	1.56	1.56	1.56	50	50	25
	CM	12.5	12.5	12.5	100	100	50
	MP	3.12	6.25	3.12	50	50	25
	TT	N/D	N/D	N/D	N/D	N/D	N/D
	KD	1.56	1.56	1.56	50	25	25
	PL	50	25	25	100	100	100

The concentration of all plant extracts was varied between the range of 100 to 0.78 mg/mL by two-fold dilution in a 96-well plate. N/D, not determined.

## Data Availability

Data sharing is not applicable.

## Sample Availability

Samples are available from the author now.
